# A novel pyroptosis-related signature predicts prognosis and response to treatment in breast carcinoma

**DOI:** 10.18632/aging.203855

**Published:** 2022-01-27

**Authors:** Haochen Yu, Yong Fu, Zhenrong Tang, Linshan Jiang, Chi Qu, Han Li, Zhaofu Tan, Dan Shu, Yang Peng, Shengchun Liu

**Affiliations:** 1 Department of Endocrine and Breast Surgery, The First Affiliated Hospital of Chongqing Medical University, Chongqing, China; 2 Medical Faculty of Ludwig-Maximilians-University of Munich, University Hospital of LMU Munich, Munich, Germany; 3 Department of Breast Surgery, Dianjiang People’s Hospital of Chongqing, Chongqing, China

**Keywords:** pyroptosis, breast cancer, immunotherapy, chemotherapy, signature

## Abstract

Background: Pyroptosis is a new form of programmed cell death (PCD), also known as cellular inflammatory necrosis. Its discovery has resulted in a novel approach to the progression and medication resistance of breast cancer (BC). However, there is still a significant gap in the investigation of pyroptosis-related genes in BC.

Methods: The mRNA expression profiles and clinical data of BC patients were obtained from the Gene Expression Omnibus (GEO) and The Cancer Genome Atlas (TCGA) databases. Then, using the TCGA cohort, we created a predictive multigene signature including pyroptosis-related genes and verified it using the two GEO cohorts. A pyroptosis-related gene signature was created by combining several bioinformatics and statistical methodologies to predict patient prognosis and responses to immunotherapy and chemotherapy. Furthermore, a nomogram based on the gene signature and clinicopathological markers was created to better classify the risk and quantify the risk assessment of individual patients.

Results: A pyroptosis-related gene signature consisting of 15 genes was established. The pyroptosis-related gene signature classified the patients into two groups: high-risk and low-risk. When combined with clinical variables, the risk score was discovered to be an independent predictor of overall survival (OS) in BC patients. Some immunological pathways and genes were linked to pyroptosis, according to Gene Ontology (GO) and Kyoto Encyclopedia of Genes and Genomes (KEGG) evaluations. Patients in the high-risk group had a worse prognosis and were not very sensitive to immunotherapy. However, several chemotherapeutic agents were predicted to have greater potential for patients in the high-risk group. Finally, a nomogram was developed that included a classifier based on the 15 pyroptosis-related genes, tumor stage, age, and histologic grade. This nomogram demonstrated good classification capacity and might help with clinical decision-making in BC.

## INTRODUCTION

According to the latest Global Cancer Statistics 2020 report, over 2,200,000 female breast cancer (BC) cases are diagnosed each year, ranking first in new cases of all tumors. With 685,000 deaths, BC is the fifth leading cause of cancer mortality globally. BC accounts for 1 in every 4 cancer diagnoses and 1 in every 6 cancer deaths in women, ranking first in incidence in the great majority of nations (159 of 185) [1]. Because of the heterogeneity of BC, early diagnosis and advancements in therapy are critical. Currently, the treatment of BC is mainly based on surgical resection, systemic chemotherapy and endocrine therapy [2]. The present effectiveness of immune checkpoint antagonists in other solid cancers has rekindled interest in immunotherapy-based BC treatment and prevention [3–5]. However, pablizumab and atezolizumab are the only currently FDA-approved immunotherapeutic agents for application in the clinical treatment of BC. Considering the limitations of BC treatments and the heterogeneity of BC, a new therapeutic target is urgently needed to improve the clinical outcome of BC; thus, a reliable novel prognostic model is also needed to make targeted therapies more feasible.

A growing number of studies have described new mechanisms of programmed cell death in recent years, including cell swelling, autophagy, ferroptosis, and pyroptosis. The most notable of these procedures is pyroptosis. In 2001, Cookson BT and Brennan Ma proposed the pyroptotic cell death mechanism as a new form of caspase-1-dependent programmed cell death for the first time [6]. Pyroptosis is a specific kind of proinflammatory cell death program that differs from other types of cell death. In particular, pyroptosis is distinguished by gasdermin family-mediated pore formation and subsequent cellular lysis, as well as the release of multiple proinflammatory intracellular cytokines [7]. In terms of its mechanism, researchers mainly believe that there are canonical pathways and noncanonical pathways involved in pyroptosis. In canonical pathways, pyroptotic processes have been proven to involve the activation of typical caspase-1 [8]. Pyroptosis is also well preserved in noncanonical pathways that involve the activation of atypical caspase-4/5/11 (human caspase-4/5 and mouse caspase-11) [8]. Pyroptosis has been detected in a number of solid tumors, indicating that it might be a possible target for solid tumor treatment [9].

Research on the relationship between BC and pyroptosis is also gradually increasing. Recent studies have shown that NLRP3 inflammasome-mediated cell death occurs in BC [10]. The tumor suppressor DRD2 has been shown to trigger pyroptosis in BC [11]. Some common chemotherapeutic agents, such as cisplatin, have also been found to induce pyroptosis in BC [12]. In fact, the role of pyroptosis in BC and other types of cancers is complicated. Recent studies have indicated that inducing pyroptosis in BC cells has opposite effects on tumor growth in two mouse breast tumor models, although they focused on different gasdermins [13, 14]. Meanwhile, because no pyroptosis-related drugs have been approved for clinical use, there are no more studies or data to clarify the relationship between pyroptosis and BC outcomes. There are also no predictive models available for BC.

Current predictions about the prognosis of BC patients are still mainly focused on the molecular level. This is most evident in the differential expression of the estrogen receptor (ER), progesterone receptor (PR), and human epidermal growth factor receptor 2 (HER2) (HER2). These conventional variables, however, are insufficient for making appropriate treatment decisions, and as a result, numerous molecular tests based on multiple gene expression patterns have been created to better predict the prognosis and treatment responses of BC patients. The most commonly used clinical gene prediction models for BC are 21-gene and 26-gene prediction models [15, 16]. It is therefore crucial to accurately profile the prognostic genes of BC, and exploring the genetic profiles associated with BC is also important for the precise treatment of BC.

Based on the available findings and data, we sought to confirm whether pyroptosis is associated with the prognosis of BC. Furthermore, we hoped to develop a pyroptosis-related signature to determine the prognosis of BC patients. The most valuable aspect of a predictive model is that it provides more information for the selection of clinical treatment options. For example, the 21-gene test in breast cancer is now guiding clinical practice [15, 17]. Therefore, we also hope that our model can provide more reference advice in the treatment of tumors (including immunotherapy and chemotherapy). This will provide a stronger theoretical foundation for the precise management of BC patients. Finally, we tried to assess the connection between pyroptosis and immunotherapy and chemotherapy in anticipation of discovering more new treatment strategies.

## RESULTS

### Schematic diagram of the study design

**Figure 1:**
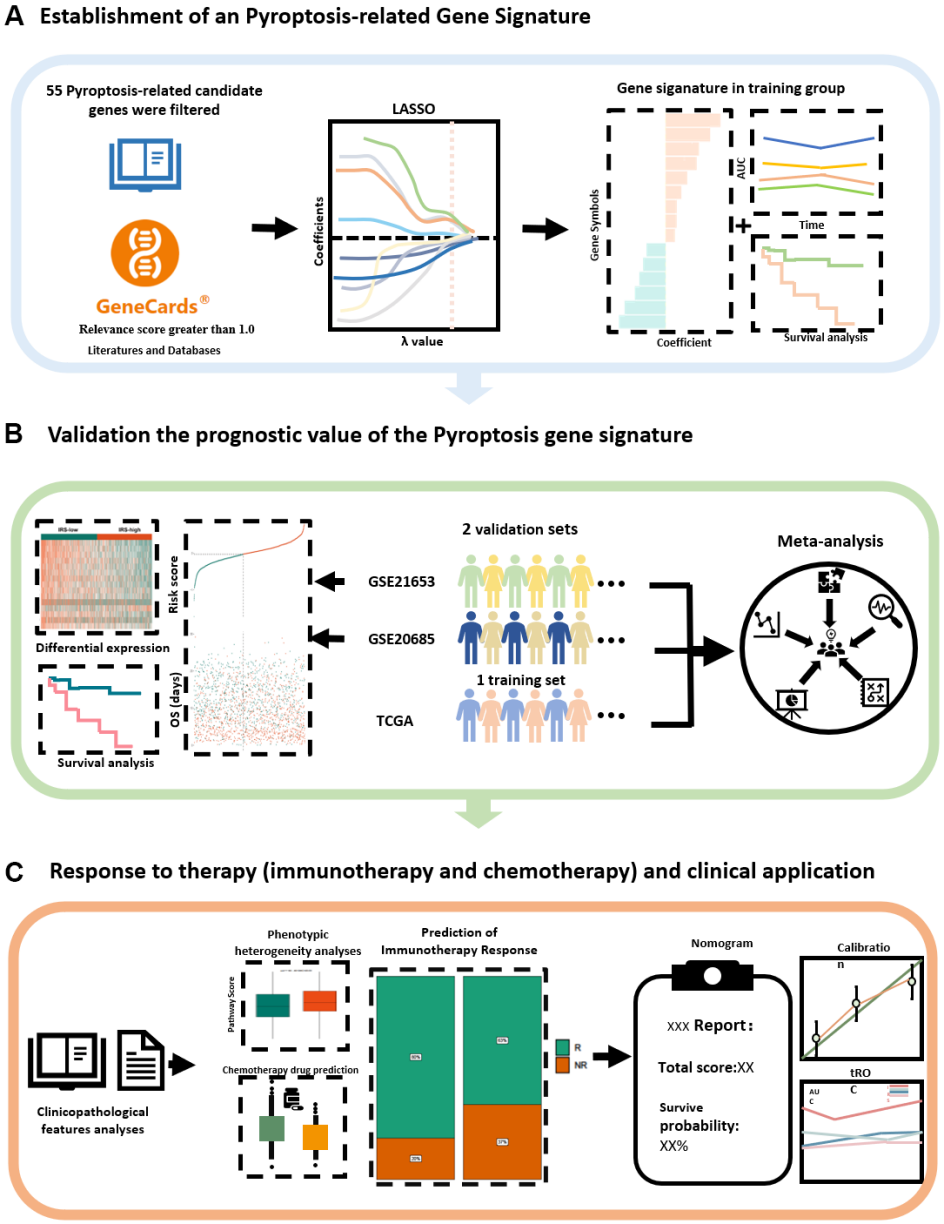
**Schematic diagram of the study design.** (**A**) A new prognostic model related to pyroptosis has been established from the literature and databases. (**B**) The stability and generalisability of the model was verified in an external independent database. (**C**) Prediction and application of PRS for clinical response to treatment. LASSO, least absolute shrinkage and selection operator; PRS, Pyroptosis-related Risk Scores; AUC, area under the curve.

Fifty-two candidate pyroptosis-related genes with relevance scores greater than 1.0 were identified from the GeneCards database, and CASP8, GZMB and ZBP1 were identified from the literature, for a total of 55 candidate genes to be finalized (Supplementary Table 1). The theory of this relevance score is explained at https://www.elastic.co/guide/en/elasticsearch/guide/current/scoring-theory.html. To identify potential candidates and create a robust signature encompassing 15 pyroptosis-related genes to predict survival, the least absolute shrinkage and selection operator (LASSO) method was utilized (Figure 1A). Subsequently, we validated the model from The Cancer Genome Atlas (TCGA) dataset as the training group in two GSE datasets as validation groups. To further confirm the model’s predictive capability, a meta-analysis was performed on the three datasets (Figure 1B). In addition to assessing the prognosis of patients, we hope that our predictive model will be useful as a clinical guideline. We therefore analyzed the relationship between clinicopathological features and the pyroptosis risk score (PRS) and explored the relationship between PRS and immunotherapy. For patients with different risks, according to our model, we also predicted several different chemotherapeutic agents that could serve as clinical treatment options. Finally, we established a nomogram to evaluate and quantify the prognosis of BC patients based on PRS and other clinical characteristic variables (Figure 1C).

### Development of a pyroptosis-related gene signature for prognosis prediction

**Figure 2:**
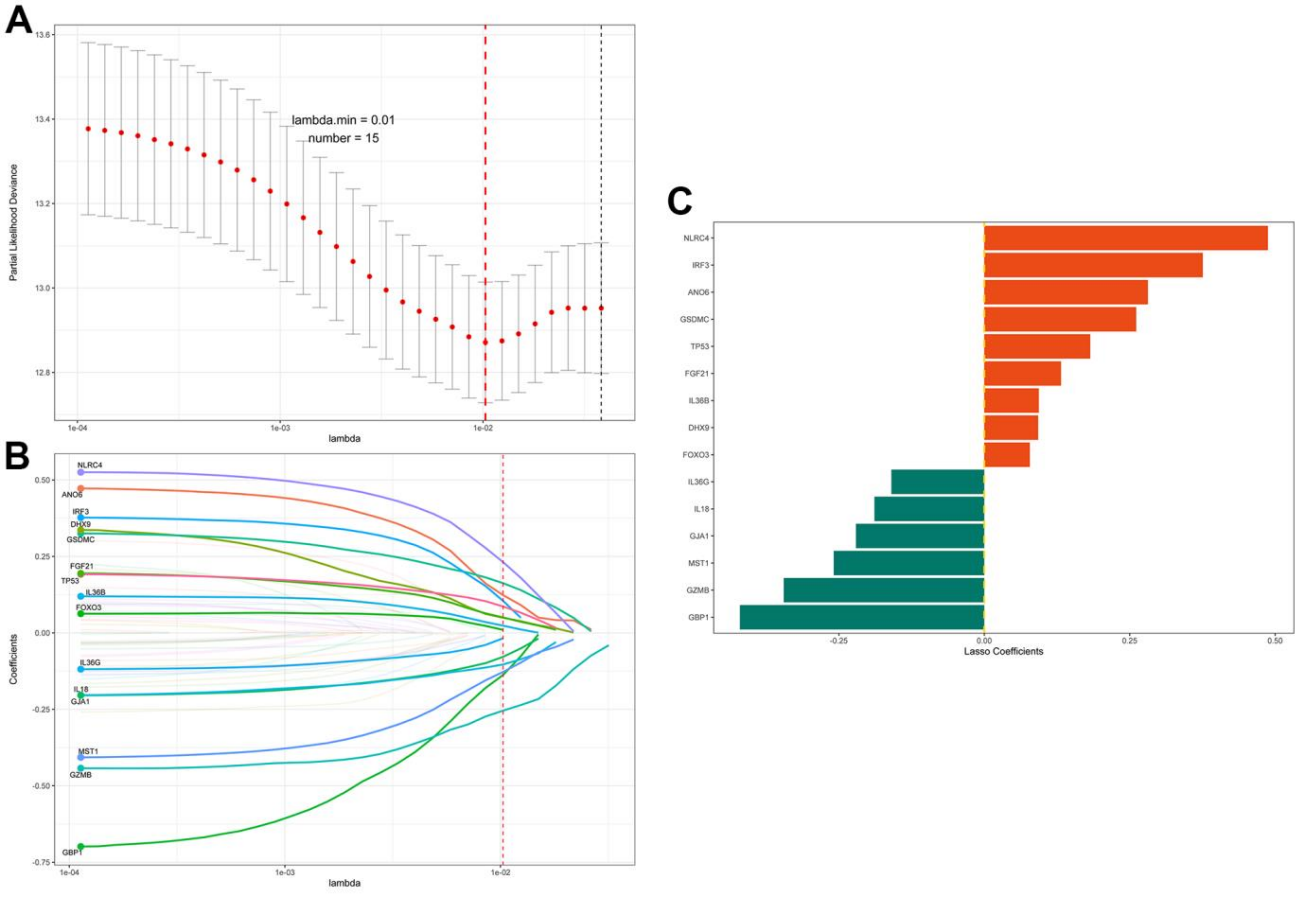
**LASSO regression identifies prognosis-related genes.** (**A**) The adjustment parameter (lambda) in the LASSO model was selected for 10-fold cross-validation by the minimum criterion. Partial likelihood deviation curves were plotted against lambda. Dotted vertical lines were drawn at the optimal values by using the minimum criterion and 1 standard error of the minimum criterion (1-SE criterion). (**B**) LASSO coefficient profiles of the 55 pyroptosis-related genes. A coefficient profile plot was produced against the log (lambda) sequence. A vertical line was drawn at the value selected using 10-fold cross-validation, where the optimal lambda resulted in 15 nonzero coefficients. (**C**) Distribution of the LASSO coefficients of the 15 immune-related gene signatures. The horizontal coordinate indicates LASSO coefficients, genes with negative coefficients in this regression indicate prognostic protective genes (green marker), and positive numbers indicate poor prognostic genes (red marker).

**Figure 3:**
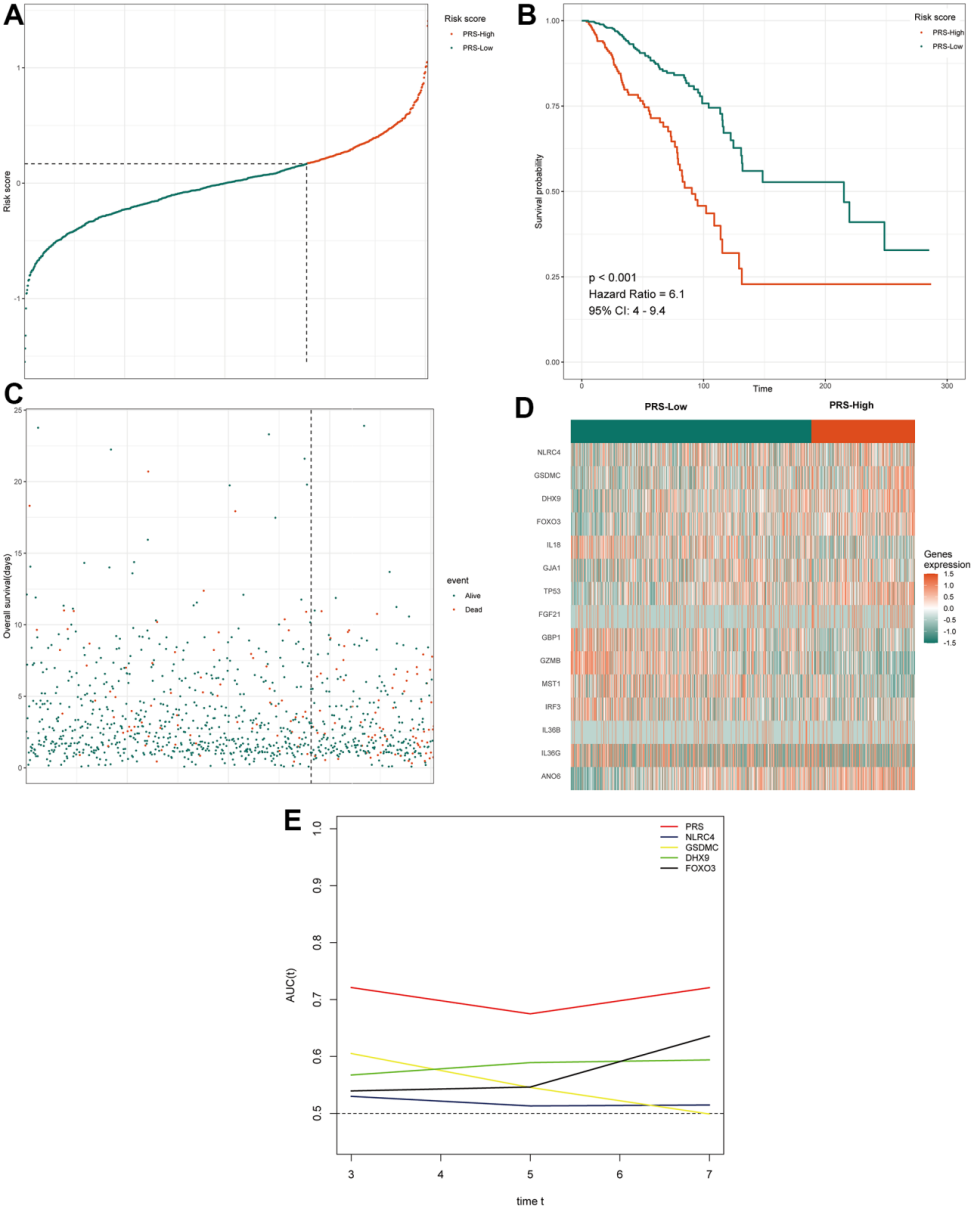
**Prognostic analysis of the 15 pyroptosis-related gene signature model in TCGA cohort.** (**A**) The distribution and cutoff value of the risk score in the TCGA cohort. (**B**) Kaplan–Meier curves for the OS of patients in the two groups in the TCGA cohort. (**C**) The distributions of OS status, OS and the risk score in the TCGA cohort. (**D**) Heatmap showing differences in the expression of 15 pyroptosis-related genes between high-PRS group and low-PRS group. (**E**) tROC analysis showed that the PRS was an accurate variable for survival prediction. The four genes shown in the figure have the four highest AUCs among the 15 signature genes. TCGA, The Cancer Genome Atlas; PRS, pyroptosis-related risk score; High-PRS, high pyroptosis-related risk score; Low-PRS, low pyroptosis-related risk score; OS, overall survival; tROC, time-dependent receiver operating characteristic; AUC, area under the curve.

To identify the genes among these 55 candidate genes that are most relevant to the prognosis of BC patients, the LASSO Cox regression model was used to identify genes with the greatest prognostic value. To overcome overfitting, tenfold cross-validation was used, with 0.01 selected as the best λ value (Figure 2A). Finally, 15 predicted genes (NLRC4, IRF3, ANO6, GSDMC, TP53, FGF21, IL36B, DHX9, FOXO3, IL36G, IL18, GJA1, MST1, GZMB and GBP1) were identified to have nonzero LASSO coefficients and were incorporated into the gene signature model (Figure 2B). Figure 2C depicts the LASSO coefficient distribution for the genes in the signature. To evaluate the prediction effectiveness of the 15 pyroptosis-related gene-based signatures in the TCGA cohort, a risk score was developed (Supplementary Table 2). Patients with a PRS greater than 0.168 were classified as high-risk, whereas those with a PRS less than 0.168 were classified as low-risk. Figure 3A, 3C show the distributions of risk scores, survival time, and survival status. As shown in Figure 3B, patients with high and low PRSs have very different prognoses. Patients with high-risk scores had a significantly shorter survival time (P < 0.001; hazard ratio (HR) = 6.1; confidence interval (CI) = 4-9.4). The expression of the 15 genes in the gene signature was examined further and is depicted in a heatmap (Figure 3D). The heatmap showed that there were differences in the expression of some of the predicted genes between the two groups. Then, the area under the curve (AUC) was calculated using time-dependent receiver operating characteristic (ROC) curve analysis. The AUCs of the signature for predicting overall survival (OS) at 3, 5 and 7 years reached 0.7211, 0.6748 and 0.7208, respectively. We selected four of the 15 characterized genes with the highest AUCs for ROC analysis and calculated the AUCs of the four genes and PRS (Figure 3E). This also suggested that, when compared to a single gene, the PRS was the most reliable predictor of OS. We examined the relationships of the PRS with clinicopathological characteristics and clinical data to investigate the link between the PRS and clinical data. PRS was discovered to be substantially related to age, tumor stage, histologic subtype, and histologic grade (Table 1).

**Table 1:** Baseline characteristics of the patients in different risk groups.

	**Low (N=699)**	**High (N=292)**	**P-value**
Age			
Mean (SD)	57.8 (12.8)	60.1 (14.1)	0.015
Median [Min, Max]	58.0 [26.0, 90.0]	61.0 [29.0, 90.0]	
Menopausal_State			
Post	447 (63.9%)	194 (66.4%)	0.5
Pre	252 (36.1%)	98.0 (33.6%)	
His_Subtype			
Ductal/NST	482 (69.0%)	229 (78.4%)	0.003
Other	217 (31.0%)	63.0 (21.6%)	
Histologic_Grade			
NA	699 (100%)	292 (100%)	<0.001
Tumor_Stage			
I	125 (17.9%)	47.0 (16.1%)	0.013
II	406 (58.1%)	162 (55.5%)	
III	155 (22.2%)	67.0 (22.9%)	
IV	6.00 (0.9%)	11.0 (3.8%)	
Missing	7.00 (1.0%)	5.00 (1.7%)	
ER_Status			
NA	1.00 (0.1%)	1.00 (0.3%)	0.073
Negative	131 (18.7%)	74.0 (25.3%)	
Positive	536 (76.7%)	210 (71.9%)	
Missing	31.0 (4.4%)	7.00 (2.4%)	
PR_Status			
NA	2.00 (0.3%)	1.00 (0.3%)	0.01
Negative	191 (27.3%)	110 (37.7%)	
Positive	474 (67.8%)	174 (59.6%)	
Missing	32.0 (4.6%)	7.00 (2.4%)	
HER2_Status			
Negative	477 (68.2%)	202 (69.2%)	0.382
Positive	100 (14.3%)	51.0 (17.5%)	
Missing	122 (17.5%)	39.0 (13.4%)	
OS.time			
Mean (SD)	44.4 (40.2)	38.2 (34.0)	0.014
Median [Min, Max]	31.8 [1.03, 285]	25.5 [1.03, 287]	
OS			
Alive	631 (90.3%)	225 (77.1%)	<0.001
dead	68.0 (9.7%)	67.0 (22.9%)	

In summary, low-risk patients are more prone to pyroptosis, or pyroptosis activity is greater in the low-risk group; that is, low-risk patients survive longer because the cancer tissue in their bodies displays higher levels of pyroptosis, resulting in more cancer cells dying. However, it is worth noting that such results do not mean that the differential expression of several genes in BC induces or suppresses pyroptosis because some of the 15 genes in this signature are not differentially expressed genes.

### Validation of the 15 pyroptosis-related gene signatures in the Gene Expression Omnibus (GEO) cohort

**Figure 4:**
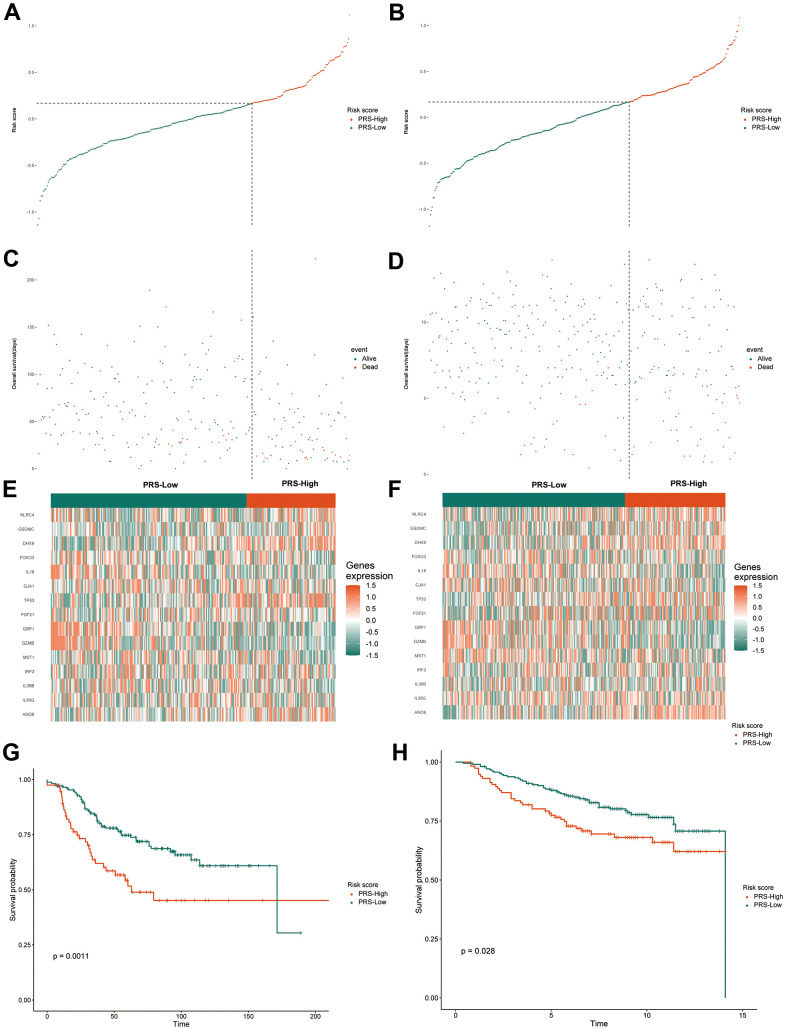
**Validation of the gene signature in the external validation sets.** The distribution and cutoff value of the risk score in the groups in the GSE20685 (**A**) and GSE21653 (**B**). The distributions of OS status, OS and the risk score in the GSE20685 (**C**) and GSE21653 (**D**). The expression heatmap of the 15 pyroptosis-related prognostic genes between the high and low-PRS groups in the GSE20685 (**E**) and GSE21653 (**F**). Kaplan-Meier curves for the OS of patients in the high-risk group and low-risk group in the GSE20685 (**G**) and GSE21653 (**H**) cohorts. TCGA, The Cancer Genome Atlas; GSE, gene expression omnibus series; OS, overall survival; PRS, pyroptosis-related risk score; High-PRS, high pyroptosis-related risk score; Low-PRS, low pyroptosis-related risk score.

**Figure 5:**
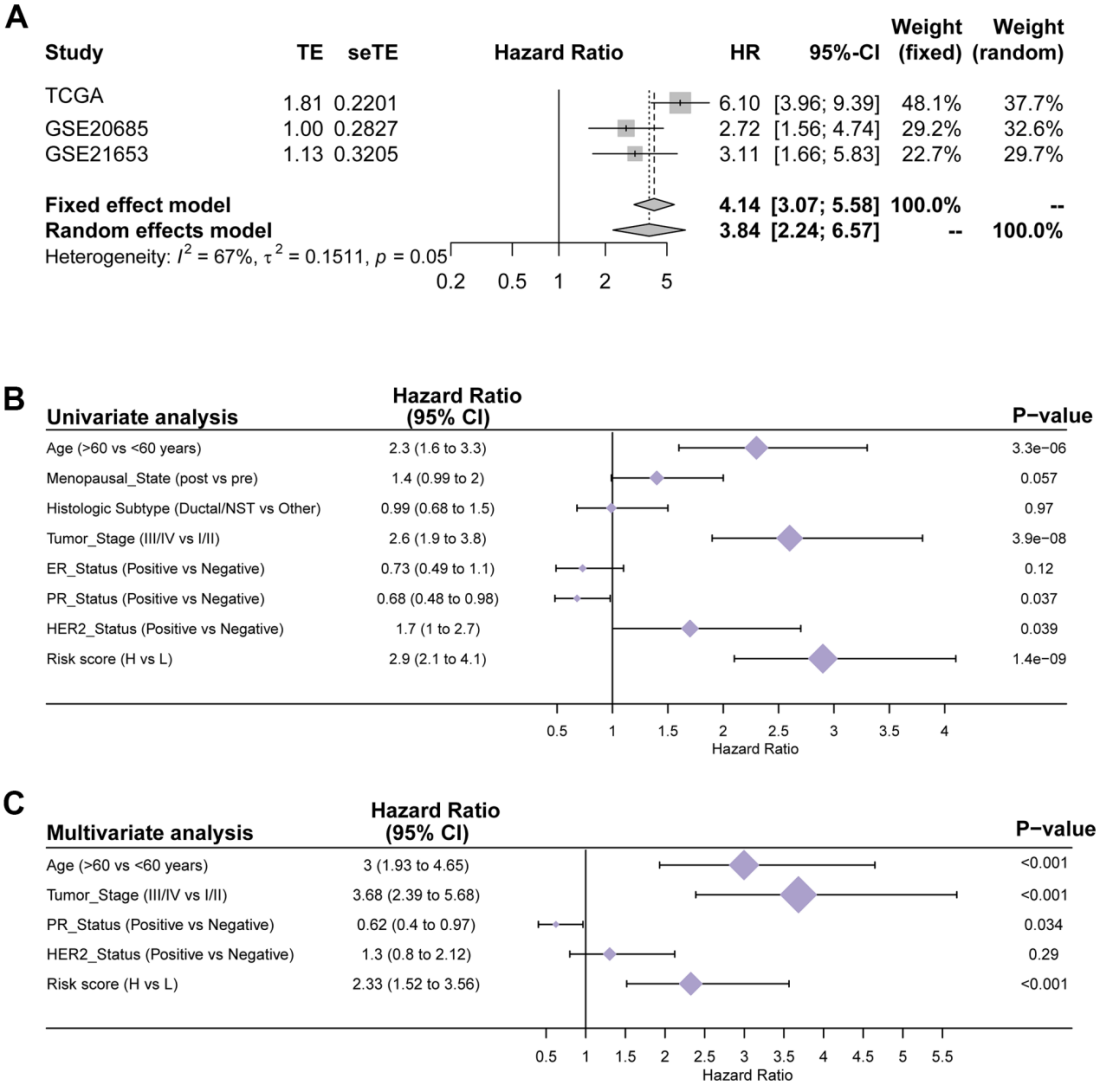
**Verification of the generalizability and stability of the prediction model in TCGA cohort.** (**A**) Meta-analysis of the TCGA training set and 2 external validation sets. (**B**) Univariate Cox regression analyses of OS in the TCGA training cohort. (**C**) Multivariate Cox regression analyses of OS in the TCGA training cohort.

The pyroptosis-related gene signature was subsequently validated in two separate external validation sets to establish its stability and generalizability in various populations. We selected two GSE datasets from the GEO database as validation sets. Patients in the GSE21653 and GSE20685 cohorts were also assigned to one of two groups: high-PRS or low-PRS. The assign formula is the same as in the TCGA cohort. The cutoff value used in both validation cohorts was 0.168, which was consistent with that used in the training cohort. Similar to the training group, in both validation cohorts, we also analyzed the distributions of the risk score (Figure 4A, 4B) and survival status (Figure 4C, 4D). The results were highly consistent with the TCGA group. The gene expression heatmaps are shown in Figure 4E, 4F. Both external validation sets’ analytical results were very consistent with those of the training set. In the two validation cohorts, Kaplan–Meier analysis revealed that low PRS predicted better OS than high PRS. (P =0.0011, Figure 4G; P =0.028, Figure 4H). Next, we conducted a meta-analysis of genetic variables in the training cohort as well as two validation cohorts separated into two groups. The meta-analysis found that all patients with greater PRS had a poorer prognosis than those with lower PRS, as illustrated in Figure 5A. (fixed effect model overall HR = 4.14, 95% CI 3.07–5.58; random effect model HR = 3.84, 95% CI 2.24–6.57). The meta-analysis showed medium heterogeneity, and we attribute this to the fact that the TCGA cohort contains a much larger number of cases than the two validation cohorts. The results of univariate and multivariate Cox regression analyses in the TCGA database are depicted using forest plots (Figure 5B, 5C), and the full data are given in Supplementary Table 3. In the TCGA cohort, the risk score was discovered to be an independent prognostic predictor. (HR=2.9, CI 2.1–4.1, P < 0.01; HR=2.33, CI 1.52–3.56, P < 0.01). Furthermore, in both cohorts, tumor stage and age were independent prognostic indicators (P < 0.01). These results demonstrate the high stability and generalizability of the 15 pyroptosis-associated gene prediction model in BC patients.

### Functional and heterogeneity analyses of the 15 pyroptosis-related genes in the high- and low-PRS groups

**Figure 6:**
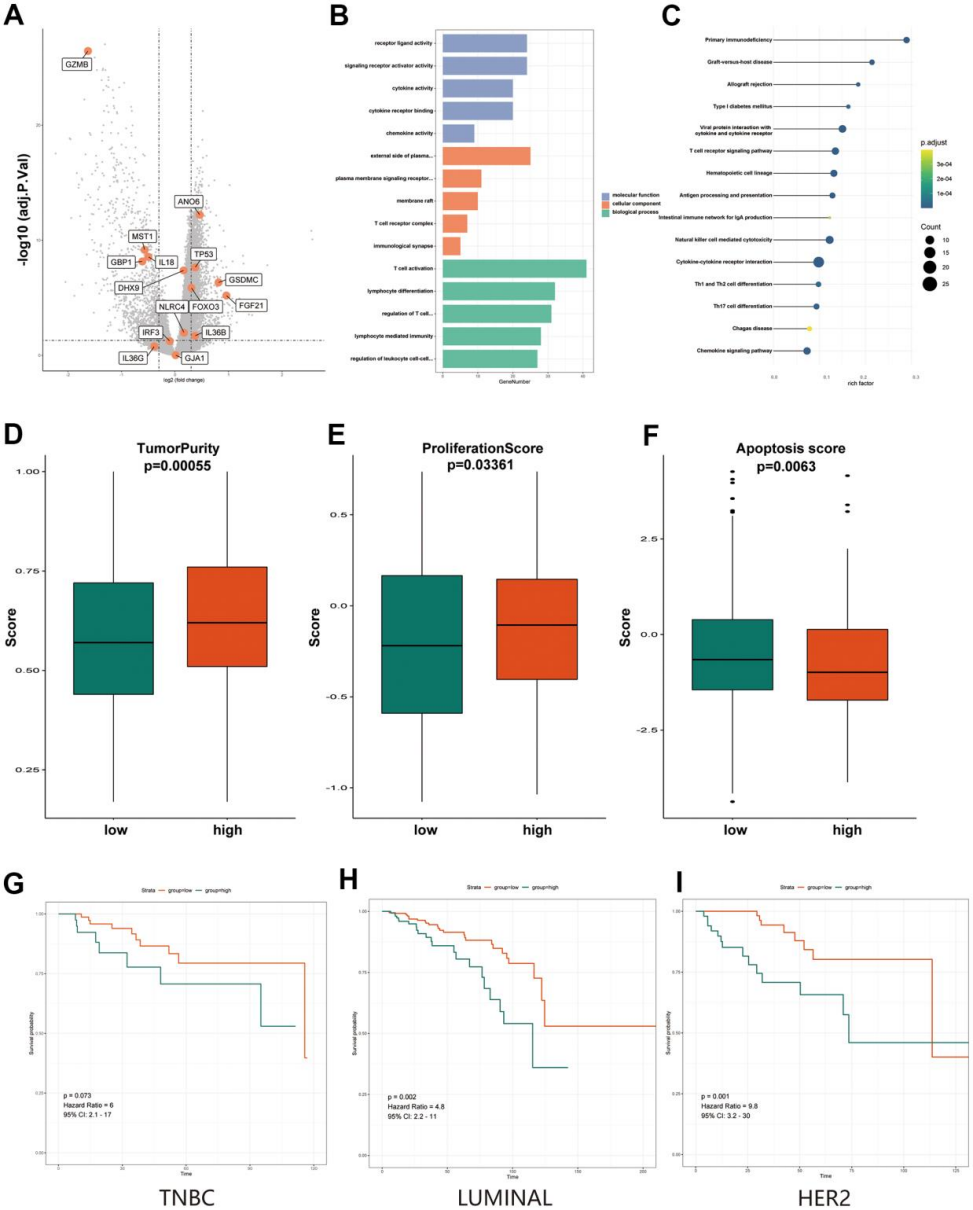
**Functional and heterogeneity analysis of 15 pyroptosis-related genes between high- and low-PRS groups.** (**A**) Volcano plot of differentially expressed genes between the low- and high-risk groups. Orange indicates the 15 pyroptosis-related gene signature. (**B**) The most significant or shared GO enrichment terms in the TCGA cohort. (**C**) The most significant or shared KEGG pathways in the TCGA cohort. (**D**) The Boxplots show differences in tumor purity between the high and low-PRS group. (**E**) The Boxplots show differences in proliferation score between the high and low-PRS group. (**F**) The Boxplots show differences in apoptosis score between the high and low-PRS group. (**G**) Results of survival analysis of high- and low- risk groups in TNBC breast cancer patients. (**H**) Results of survival analysis of high- and low- risk groups in LUMINAL breast cancer patients. (**I**) Results of survival analysis of high- and low- risk groups in HER2 breast cancer patients. The Kruskal–Wallis test was performed to calculate the P-value. GO, Gene ontology; KEGG, Kyoto Encyclopedia of Genes and Genomes; PRS, pyroptosis-related risk score; High-PRS, high pyroptosis-related risk score; Low-PRS, low pyroptosis-related risk score.

To determine exactly what role these genes play in terms of their expression and function, further analyses are needed. We analyzed the expression and functions of these 15 pyroptosis-related genes. Some pyroptosis-related genes, including ANO6, TP53, GSDMC, FGF21 and IL36B, had high expression levels in BC patients (Figure 6A). We also found some genes that were not significantly different in expression, including DHX9, NLRC4, IRF3 and GJA1 (Figure 6A). We believe that these genes, which are not significantly differentially expressed but have an impact on prognosis, play a part in the regulation of certain functions. GO and KEGG pathway analyses were also utilized to investigate the probable functions of these genes in the two groups. Interestingly, the TCGA cohort’s pyroptosis-related genes were shown to be enriched in various cancer-related molecular pathways, including immune-related pathways such as T cell activation, T cell regulation, and primary immunodeficiency (Figure 6B, 6C). Reversed-phase protein arrays (RPPAs) were utilized to assess the key pathways in the two groups to further explore tumor heterogeneity between the two groups of patients and to examine the variations in tumorigenesis processes between the two groups. The analysis results showed that tumor purity, proliferation scores, and apoptosis scores differed between the high- and low-risk groups (Figure 6D–6F). All three scores may indicate that high-risk patients have more malignant tumor cells that are prone to metastasis and have more difficulty undergoing apoptosis. The pathway scores, which are protein expression signatures of pathway activity, associated with tumor lineage were from an RPPA in prior work in the literature [18]. Given the highly heterogeneous character of breast cancer, the prognosis of different subtypes of BC patients differs considerably. As a result, we investigated the prognosis of patients in various risk categories among different subtypes of BC patients. The results showed that in patients with HER2 and LUMINAL subtypes, the low-risk group had a better prognosis than the high-risk group (Figure 6H, 6I), while the prognosis of patients with triple-negative breast cancer (TNBC) was not (Figure 6G).

### Response to chemotherapy and immunotherapy of high- and low-PRS patients

**Figure 7:**
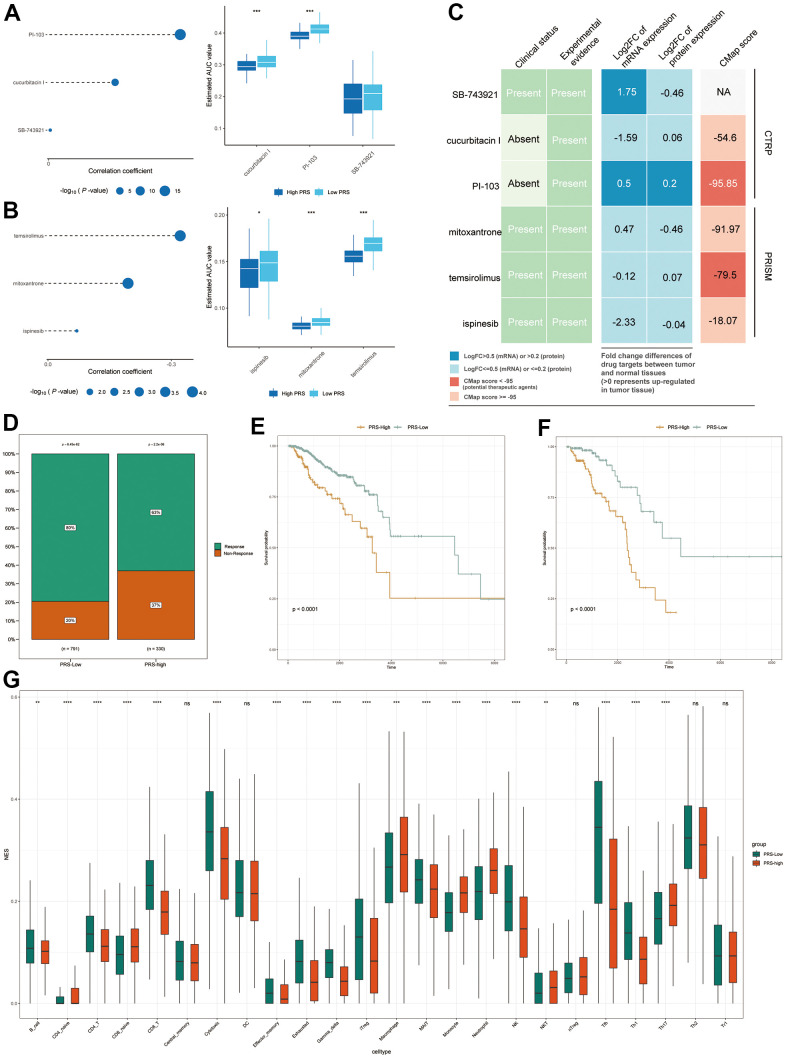
**Response to chemotherapy and immunotherapy of high- and low-IRS patients.** (**A**, **B**) The bubble plot shows the degree of negative correlation between AUC values and PRS scores of three CTRP-derived compounds (**A**) and three PRISM-derived compounds (**B**). Longer lines in the graph indicate a stronger negative correlation, predicting greater drug sensitivity. Boxplots indicate the results of differential drug response analysis between the PRS high and PRS low groups for the three CTRP-derived compounds (**A**) and the three PRISM-derived compounds (**B**). Note that lower values on the y-axis of boxplots imply greater drug sensitivity. (**C**) Evidence from multiple sources to identify the most promising therapeutic agents for the high-IRS group. Six compounds come from CTRP and PRISM are shown on the diagram, respectively. (**D**) Proportion of immune-responsive and nonimmune-responsive populations in the PRS high- and low-expression groups. (**E**, **F**) Survival analysis graph showing differences in survival between PRS-high and IRS-low groups in nonimmune-responsive (**E**) and immune-responsive (**F**) patients. (**G**) Differences in immune infiltration scores of 24 immune cell types in high and low PRS groups in TCGA database. * means p <0.05, ** means p <0.01, *** means p<0.005, **** means p<0.001.

Systemic chemotherapy remains the foundation of BC treatment. However, some refractory, advanced tumors lack more chemotherapy options. Therefore, we used two chemotherapy drug response databases (PRISM and CTRP) to identify new highly sensitive chemotherapy candidates for high PRS patients. First, the AUC values and PRSs were analyzed by Spearman correlation analysis to identify chemicals with negative correlation coefficients (Spearman r < -0.01 for CTRP and -0.01 for PRISM). Next, an examination of drug response differences between the high- and low-PRS groups was performed to find drugs with lower estimated AUC values in the high-PRS group [log2-fold change (FC) >0.07]. It is essential to note that a lower AUC indicates higher drug sensitivity. Three CTRP-derived compounds (cucurbitacin-I, PI-103 and SB743921) and three PRISM-derived compounds (including temsirolimus, mitoxantrone and ispinesib) were found to be potentially sensitive in the high-PRS group. In the high-PRS group, all of these drugs displayed a negative connection with the PRS and lower calculated AUC values. (Figure 7A for CTRP and Figure 7B for PRISM). For the chemotherapy drug analysis, we also analyzed the responses to some of the known and widely used chemotherapy drugs, cisplatin and fluorouracil. The results showed that these two common chemotherapy drugs also differed significantly in high- and low-risk populations. The low-risk populations had lower AUC values, indicating greater sensitivity to the two common chemotherapeutic agents. (Supplementary Figure 1). Multiple-perspective studies were undertaken to assess the therapeutic potential of these compounds in BC to further evaluate whether the compounds we found had potential clinical utility. First, a detailed assessment of the literature in PubMed was conducted to discover experimental and clinical evidence of the six prospective medications in the treatment of BC. Second, the log2 FC values of the differential mRNA and protein expression levels of genes relevant to drug targets between tumor and normal tissues were determined, and a larger log2 FC value suggested better potential for BC therapy. Third, CMap analysis was utilized to confirm compounds whose gene expression patterns were oppositional to the BC-specific expression patterns (the expression of some genes was raised in tumor tissue but lowered after treatment with particular drugs). Lower CMap scores imply that these chemicals might have therapeutic potential for BC (Figure 7C). The approaches described above are based on published literature [19].

In addition to chemotherapy, immunotherapy is a new direction for BC treatment. Additionally, in our previous GO and KEGG analyses, we found that these genes appear to be inextricably linked to the immune system. Therefore, we sought to determine whether there was a difference in the response to immunotherapy between the high- and low-risk groups. Figure 7D depicts the predicted response to immunotherapy of patients with high and low PRS. In the high-PRS group, only 63% of patients were expected to react to immunotherapy, but in the low-PRS group, this proportion was 80%. Based on these findings, we hypothesize that patients with low PRS may be more susceptible to immunotherapy. Patients with a high PRS had a poorer prognosis in both groups when survival analysis was performed individually in the immunotherapy-responsive and immunotherapy-nonresponsive groups (Figure 7E, 7F). These findings imply that patients with high PRS are not especially sensitive in terms of immunological response. This may be because of the different expression of genes associated with immunity. To further explore the relationship between the PRS and immune status, the immune infiltration scores in TCGA were calculated by using the Immune Cell Abundance Identifier (ImmuCellAI), which is used to precisely assess the abundance of 24 immune cell types, including 18 T cell subsets. (Figure 7G). It is encouraging to note that most of the immune cells, especially the various types of T cells, demonstrated significant differences in the different PRS groups (adjusted P < 0.05). These findings imply that the 15 pyroptosis-related genes we selected are potentially related to immune infiltration.

### Developing a prediction nomogram for BC patients

**Figure 8:**
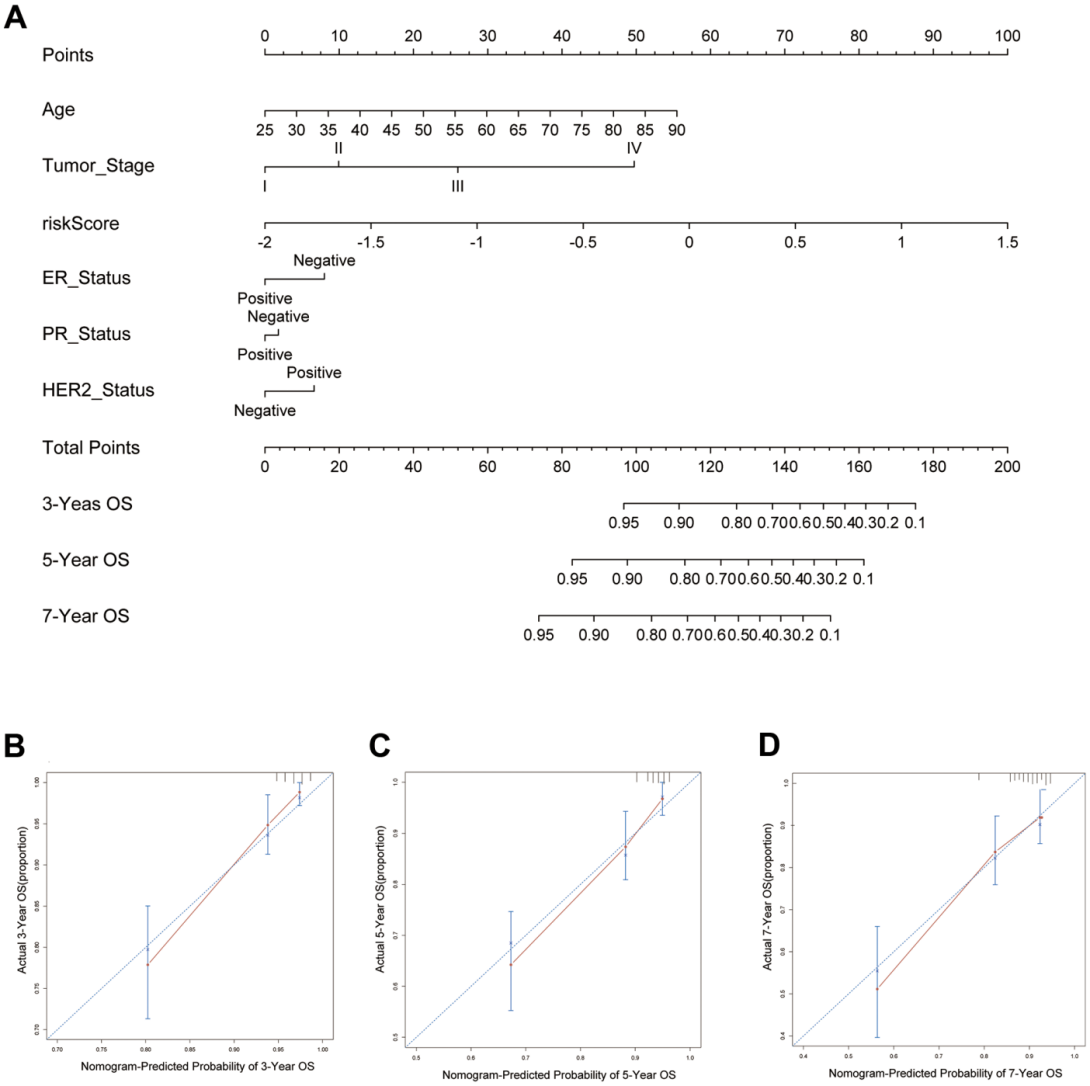
**Predictive nomogram for PRS and clinical features.** (**A**) The 15 pyroptosis-related prognostic model for predicting 3-, 5-, and 7-year OS in BC patients. The independent risk factors were used to build a risk estimation nomogram to predict the probability of OS in BC patients. (**B**) The calibration plots for 3-year survival probabilities in the TCGA cohort. (**C**) The calibration plots for 5-year survival probabilities in the TCGA cohort. (**D**) The calibration plots for 7-year survival probabilities in the TCGA cohort.

Independent risk indicators were used to develop a risk assessment nomogram to provide a clinically relevant strategy for predicting the OS probability of BC patients (Figure 8A). These indicators included age, tumor stage, PR status, HER2 status, ER status and the risk score related to pyroptosis. Figure 8B–8D show calibration plots for 3-, 5-, and 7-year survival probabilities in the TCGA cohort, indicating that the nomogram had a high degree of accuracy. This indicates that our nomogram has a high predictive value.

## DISCUSSION

Tumor development is dependent on the survival and death of tumor cells. Therefore, studying cell death can help us decipher the underlying mechanisms of tumors. In addition to the familiar methods of apoptosis and necrosis, more forms of programmed cell death are being discovered by researchers. For example, ferroptosis has been hotly studied in recent years [20]. Research on pyroptosis has also become more frequent but has mostly focused on more in-depth basic studies. Little has been reported in the literature as to whether this new mode of cell death can provide clinicians with some therapeutic insight. There are also few studies in the field of BC. Therefore, we sought to investigate the relationship between pyroptosis and BC clinical data in anticipation of obtaining more new methods that can be used for clinical diagnosis and treatment.

In our study, we first identified 55 candidate pyroptosis-related genes from the literature and the GeneCards database. LASSO Cox regression was performed to establish a new prognostic model comprising 15 pyroptosis-related genes that was validated in 2 external cohorts. Unlike many other signature articles, we did not select differentially expressed genes in BC when we identified the initial candidate pyroptosis-related genes. Many genes with significant differential expression were not predictive of the prognosis of patients. The significance of a pyroptosis-related signature is mainly to provide some suggestions for clinical work, so we still consider the prognosis of the patient as the first principle. Therefore, we included as many genes related to pyroptosis as possible when we included candidate genes. In fact, our team used both methods to make our initial candidate gene list (differential gene screening and database relevance screening). We found that the predictive power (mainly the AUC values) of the predictive models built by differential gene screening was not as good as the method we have chosen now. The final 15 genes we obtained were NLRC4, IRF3, ANO6, GSDMC, TP53, FGF21, IL36B, DHX9, FOXO3, IL36G, IL18, GJA1, MST1, GZMB and GBP1. Among our final selection of 15 genes, IL18, NLRC4, IRF3, and GJA1 were not differentially expressed genes in BC but still had a good predictive role.

As an inflammatory type of regulated cell death, the main pathological characteristics of pyroptosis are cell swelling and lysis. This process is accompanied by the release of many proinflammatory factors, including many members of the interleukin family, such as IL18 and IL1. There are two main pathways of pyroptosis activation: (i) GSDMD (gasdermin D)-dependent activation regulated by caspase 1/4/5/11 and (ii) GSDME-dependent activation regulated by caspase 3 [21–24]. The GSDM family plays a crucial role in the activation of pyroptosis. Activated caspases cleave the hinge region between the N- and C-terminal domains of GSDMD or GSDME, releasing the lethal active segment and leading to pyroptosis [25, 26]. The GSDM family is mainly responsible for pore formation during the activation of pyroptosis. GSDMC has also been reported to have a pore-formation domain and can induce pyroptosis [14, 26]. Thus, it is reasonable that IL18 and IL36 from the interleukin family and GSDMC from the GSDM family were included in our final 15 genes.

**Figure 9:**
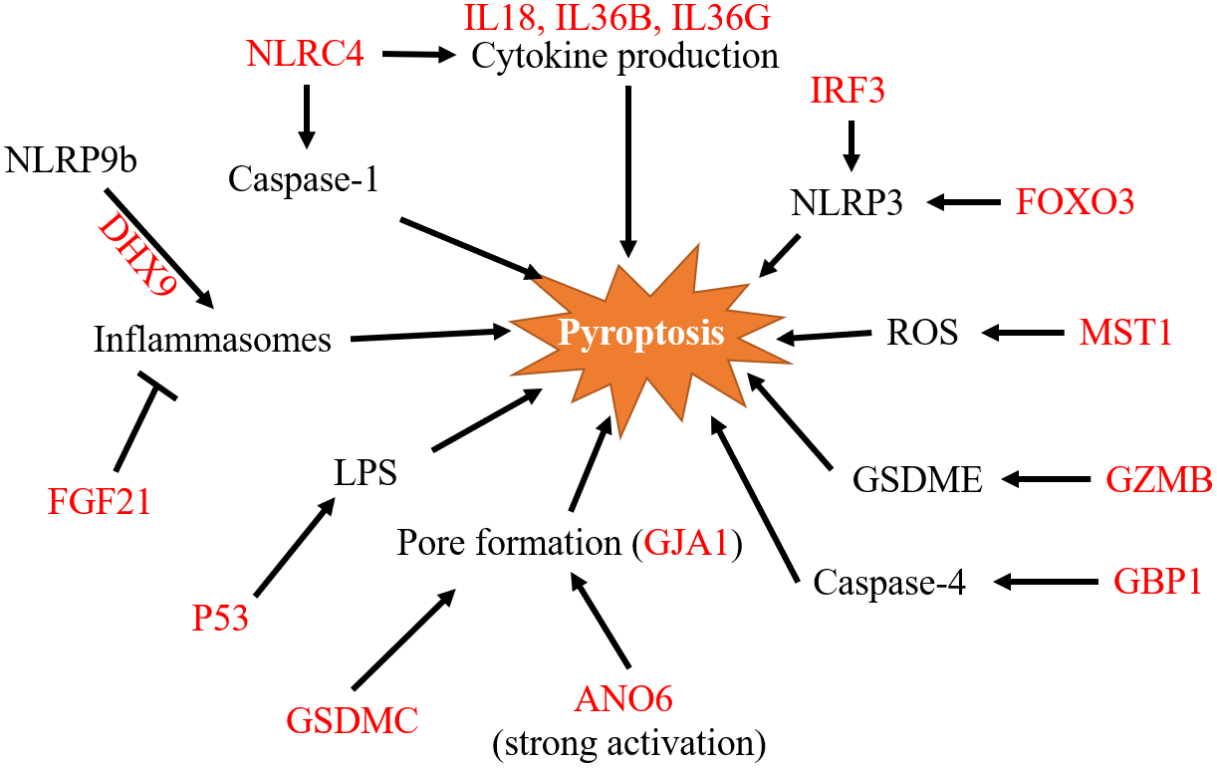
**A pathway map of the 15 pyroptosis-related signature genes.** Pathway map of 15 genes involved in pyroptosis regulation as summarized from references. ROS, reactive oxygen species; LPS, lipopolysaccharide.

We carefully analyzed the pathway of these 15 genes regulating pyroptosis and exhibited the pathway map of the 15 genes in Figure 9. The whole process of pyroptosis is accompanied by the formation of inflammasomes, so genes that affect inflammasome formation are also very likely to affect pyroptosis. NLRC4 is a key inflammasome component that indirectly detects particular proteins from pathogenic bacteria and fungi and responds by building an inflammasome complex that promotes caspase-1 activation and cytokine production. Phosphorylation of NLRC4 by PKCd and leucine-rich repeat kinase 2 (LRRK2) triggers the formation of a NOD-like receptor family apoptosis inhibitory protein (NAIP)–NLRC4 complex and the recruitment of caspase-1 to form an inflammasome [27]. IRF3, a member of the interferon regulatory family, is related to the NLRP family and has been shown to activate NLRP3 to promote pyroptosis [28]. In contrast, downregulation of FOXO3 reduces NLRP3 inflammasome-mediated endothelial cell pyroptosis in atherosclerosis [29]. Additionally, related to the inflammasome is FGF21. Existing studies support that FGF21 interferes with pyroptosis by inhibiting the formation of inflammasomes [30]. P53-specific knockdown prevented pyroptosis produced by lipopolysaccharide (LPS). *In vivo*, p53 overexpression in A549 cells significantly reduced tumor development and the death rate by raising the pyroptotic level [31]. ANO6 belongs to the anoctamin family and is a multipass transmembrane protein. ANO6 exhibits intriguing properties in that it contributes to apoptotic cell death at low levels of activation, causing cell shrinkage. However, when activated strongly, it contributes to pore production and generates significant membrane blebbing, cell swelling, and membrane disintegration [32]. Based on the morphological characteristics of pyroptosis described earlier, we hypothesize that the high expression of ANO6 can promote cellular pyroptosis. DHX9 encodes an RNA helicase. In 2017, Zhu S et al. found that via the RNA helicase Dhx9, NLRP9b recognizes short double-stranded RNA stretches and forms inflammasome complexes with the adaptor proteins Asc and caspase-1 to accelerate the maturation of IL18 and GSDMD-induced pyroptosis [33]. It has been suggested that gap junction proteins can induce pyroptosis [34]. GJA1, a gap junction protein, may also be involved.

MST1 was found to inhibit pancreatic cancer progression through ROS-induced pyroptosis in pancreatic cancer [35]. Because NK-induced pyroptosis in GSDME-expressing cells does not require caspases, GZM proteases are hypothesized to cleave GSDME. GZM-mediated cell death is caspase-independent but amplified by caspases because GZMB cleaves and activates caspase-3 [13]. GBP1 enhanced caspase-4 recruitment to Salmonella, resulting in increased activation and pyroptosis [36].

It is important to note that our study is not intended to show that the high or low differential expression of the 15 genes does or does not lead to pyroptosis. In reality, there is no obvious link between the expression of these genes and the development of pyroptosis. As in our previous analysis, not all 15 genes were directly related to pyroptosis. Meanwhile, some genes that are closely related to pyroptosis, such as GSDMD and GSDME, were not included in our signature. Our signature only demonstrates that these pyroptosis-related genes may be combined to accurately predict the survival time of BC patients.

Our research not only uncovers the link between these genes and BC prognosis but also focuses on novel therapeutic strategies for BC patients. In recent years, immune checkpoint inhibitor (ICI)-based therapies seem to have achieved some research results [37]. However, some data show that only one-third of tumor patients benefit from them, so there are some limitations to ICI treatment [38]. Although the role of pyroptosis has been confirmed in a variety of tumors, its exact relationship with the immune system is not clear. Our findings imply that patients with low PRS respond better to immunotherapy. Thus, there may be a very strong relationship between pyroptosis and immunotherapy. In 2020, Zhang et al. showed that NK cells and CD8+ T cells reciprocally induce pyroptosis in cancer cells via granzyme B, which is capable of cleaving GSDME [13]. In separate research published the same year, CD8+ T cells and NK cells were demonstrated to promote tumor clearance via the GSDMB granzyme A axis [39]. Taken together, these studies suggest that NK cells may activate pyroptosis but may have different axes in different cells. The relationship between CD8+ T cells and pyroptosis is still a focus of the GSDM family. As early as 1986, it was reported that GSDMD limits the cytolytic ability of CD8+ T cells [40]. An *in vitro* experiment also implied that GSDMD is required for the antitumor effect of CD8+ T cells [41]. Our findings also revealed that the immune infiltration capability of NK cells and CD8+ T cells differed considerably between the two groups of patients with different PRSs. In the TCGA cohort, as expected, patients with low PRS were more vulnerable to tumor immune responses than those with high PRS. Interestingly, it seems that some innate immune cells, such as macrophages, monocytes, and neutrophils, scored higher in the high-PRS group. In contrast, regulatory immune cells such as several adaptive immune cells such as CD8 T cells, B cells, CD4 T cells, etc. had higher scores in the low-PRS group. Innate immunity has a weak effect and a short duration of action. The effect of regulatory immunity remains strong and long. Innate immunity and regulatory immunity together constitute the total defense function of the body. We consider that strong and persistent regulatory immunity gives a better prognosis for patients. Although patients in the high-PRS group had seemingly high levels of innate immunity, there was little subsequent activation of important regulatory immunity, which may have contributed to poor clinical outcomes. However, exactly which pyroptosis-related genes regulate the two different immunizations requires more experiments to prove.

Therefore, we speculate that treatment targeting ICIs in combination with pyroptosis is a new potential treatment strategy. A review supported this strategy and reported that ICIs efficiently killed cold tumor cells only in the context of the concomitant induction of pyroptosis. Similarly, pyroptosis induction alone failed to trigger efficient tumor inhibition, highlighting the importance of treating cold tumors with a combination of pyroptosis inducers and ICIs [42]. Therefore, we believe that after numerous *in vivo* and *in vitro* experiments, such new therapies may provide a new pathway for tumor treatment.

Systemic chemotherapy remains the basis of the treatment of BC. However, due to the high heterogeneity of BC, the chemotherapy regimen selected varies from patient to patient. Some commonly used chemotherapy drugs, such as paclitaxel and cisplatin, have been demonstrated to limit tumor proliferation and metastasis by triggering pyroptosis [43, 44]. By our prediction of chemotherapeutic agents, we identified six agents with different effects in patients with high PRS and low PRS. These drugs are not common chemotherapeutic agents for BC, and more basic research would allow us to confirm whether they can be used in the treatment of BC. Cucurbitacin-I, a glucocorticoid agonist, has been shown to promote apoptosis and induce autophagy in a variety of tumors [45–47]. PI-103, a PI3K/mTOR inhibitor, has also been published to be related to the regulation of a variety of tumor cells [48, 49]. SB743921 is a strong inhibitor of the spindle protein kinesin that is being researched for the treatment of myeloma in ongoing clinical trials [50]. Temsirolimus and ispinesib target mTOR and EGFR and have been shown to inhibit a variety of tumors [51, 52]. Among them, temsirolimus is being studied in phase III clinical trials of triple-negative BC. Mitoxantrone is a synthetic anthracenedione that was originally created to enhance the therapeutic profile of anthracyclines. It is often used to treat breast and prostate cancers, lymphomas, and leukemias [53]. Of these six drugs, temsirolimus and mitoxantrone have been or are likely to be used in the treatment of BC. In our analysis data, patients with a high PRS were more sensitive to these two drugs. If there are more data to support this finding, the detection of PRS when using these two drugs in the future may support precision treatment.

Our signature predicted many therapeutic agents with significant differences in drug sensitivity between the high- and low-PRS groups, as expected. Of note, such drug prediction is not aimed at general low-risk BC patients with a better prognosis. Rather, it is designed to target the high-risk population with a poorer prognosis. We hope that our model will aid in the prediction of new drugs and the identification of new therapeutic targets and bring new hope to patients with a poor prognosis.

We found that some pyroptosis-related articles have been published recently. However, there are no articles related to BC. The main areas of these studies are lung adenocarcinoma [54], gastric cancer [55] and ovarian cancer [56]. In the adenocarcinoma article, the authors ultimately included only five genes to build the model. The biggest drawback is that there was no validation group to prove the reliability of the model. The article on ovarian cancer analyzed a validation group. They used differential genes to directly divide patients with ovarian cancer into two clusters and then analyzed survival. The results were not statistically significant. This supports our view that not all differentially expressed genes will be meaningful in terms of survival time. Therefore, we avoided this when we included candidate genes in the first step. Relatively speaking, the article on gastric cancer is more comprehensive, and it also has some reference significance for our research. Compared with this article, our advantage is that in addition to the analysis of immune infiltration, we also predicted potential chemotherapeutics and provided some new potential options for chemotherapeutic agents.

Nevertheless, there are still some general limitations in our study. As with most predictive modeling articles, the primary source of our data is publicly available databases, which lack the validation of laboratory data and real clinical data. Even though we used two different GSE datasets to validate our model, it still cannot replace validation with real clinical data. Therefore, the feasibility and true predictive value of the pyroptosis-related gene signature in clinical applications needs to be validated by more prospective studies.

In conclusion, our research systematically built a unique prognostic model comprised of 15 pyroptosis-related genes. The TCGA training cohort created this prognostic model, which was verified in the two GSE validation cohorts. According to our prediction model, BC patients can obtain a PRS, and according to the score, we can determine whether the patient is sensitive to immunotherapy. With this score, it is also possible to predict which chemotherapy drugs they are more sensitive to. We created a nomogram to assess the risk of BC patients by combining this information with clinicopathological factors. This model based on the pyroptosis-related gene signature could be a useful tool to facilitate the personalized management and precision treatment of cancer.

## MATERIALS AND METHODS

### Data collection

The training set consisted of the RNA data collected and clinical information from 1007 BC patients acquired from The Cancer Genome Atlas (TCGA). In addition, the GSE21653 (n=266) and GSE20685 (n=327) datasets, all of which contain gene expression files from the Gene Expression Omnibus (GEO) database, were utilized as external validation sets. Only patients with overall survival (OS) times of more than 30 days were included in all three datasets. Candidate pyroptosis-related genes were identified mostly from the literature and the GeneCards database (https://www.genecards.org/) using the term pyroptosis, and genes with relevance scores greater than 1.0 were selected. In the list of candidate genes, CASP8 [57], GZMB [58] and ZBP1 [59] were screened by reading extensive literature and were not in the Genecards database screening results. All candidate pyroptosis-related genes are provided in Supplementary Table 1. The infiltration scores of 24 immune cells were obtained from ImmuCellAI (http://bioinfo.life.hust.edu.cn/web/ImmuCellAI/) [60] and normalized in R software (version 4.0.3). ImmuCellAI is a website that estimates the abundance of 24 immune cells using gene expression datasets such as RNA-Seq and microarray data. It may be used to quantify the difference in immune cell infiltration across groups and predict patients’ responses to immune checkpoint blockade treatment. It works by obtaining a reference expression profile for each cell type from the GEO database and curating a specific gene set from a publication as a gene signature. Then, the total expression deviation of the gene signatures in the input expression profiles from the reference expression profiles of the 24 immune cell types was calculated. Deviations were then assigned to the corresponding immune cell type based on the enrichment fraction of its genetic profile, which was calculated using the single sample gene set enrichment analysis (ssGSEA) algorithm [60]. Drug sensitivity data of cancer cell lines (CCLs) were gathered from the Cancer Therapeutics Response Portal (CTRP v.2.0, released October 2015) and the PRISM Repurposing dataset (19Q4, released December 2019).

### Study design

The training set was the TCGA cohort, while the validation sets were the GSE20685 and GSE21653 cohorts. Fifty-five potential pyroptosis-related genes were found mostly through research articles and search results of the GeneCards database, with pyroptosis as the keyword, and genes with correlation scores greater than 1.0 were selected. The 55 genes were then evaluated using the R package glmnet (version: 4.0-2) for least absolute shrinkage and selection operator (LASSO) Cox regression to filter down the possible pyroptosis-related genes. The 55 pyroptosis-related genes were found to have nonzero coefficients in the model, and the samples were divided into high- and low-risk groups based on the cutoff value of 0.1677 obtained using the survminer R package’s surv cutpoint function (Version: 0.4.3). The formula of the pyroptosis risk score (PRS) was as follows:

PRS = sum of coefficients × normalized expression level of pyroptosis-related genes.

Finally, independent risk variables found by multivariate Cox regression analysis were used to create a nomogram for predicting patient OS. Finally, to evaluate the performance of the nomogram, calibration plots were created. The concordance index (C-index) was used to examine the consistency between the model’s projected probability and the actual outcomes. The R package rms was used to plot the nomogram and calibration plots (Version: 4.0.2).

### Gene set enrichment analysis (GSEA) and functional enrichment analysis of pyroptosis-related genes

GSEA was used to discover differences in the mechanisms in BC patients to analyze the potential pathways of pyroptosis-related genes. Based on the risk score, genes with P < 0.05 and |log2-fold change (FC)| ≥1 were considered to be substantially differentially expressed between the high- and low-risk groups. The clusterProfiler R tool was used to perform GO and KEGG analysis on these pyroptosis-related genes [61].

### Estimation of chemotherapy and immunotherapy responses

Data on immunotherapy responses (anti-PD1 or anti-CTLA4) were obtained from ImmuCellAI, a gene set signature-based technique developed for predicting immunotherapy responses with high accuracy by evaluating gene expression data [60]. Through this tool, we divided BC patients into immunotherapy-responsive and nonresponsive groups. The chi-square test was then used to analyze the immunotherapy response group and the PRS group. The infiltration scores of the 24 immune cells from ImmuCellAI were also used to compare the differences between the high- and low-PRS groups.

Proteomic analysis is one of the commonly used tools in tumor pathophysiology. The TCGA project team has made extensive use of reverse-phase protein array (RPPA) technology to perform tumor proteomic studies. This microarray allows analysis of the expression and trends of multiple marker proteins in the sample. By integrating RPPA microarray data from TCGA and several independent tumor research projects, The Cancer Proteome Atlas (TCPA) is provided to facilitate researchers to view and analyze the visualized microarray data. The article was published in nature methods [62]. The data we used in this study were the breast cancer RPPA microarray data compiled by Ciriello G et al. and can be downloaded directly from the supplemental data in the article [18].

The connectivity map (CMap) is a resource that uses cellular responses to perturbation to find relationships between diseases, genes, and therapeutics. The list of up- and downregulated differentially expressed genes obtained from the experimental analysis was compared with the database reference dataset using CMap; a correlation score (-100 to 100) was calculated based on the enrichment of differentially expressed genes in the reference gene expression profile; a positive number indicated that the up- and downregulated differentially expressed genes were similar to the reference gene expression profile; a negative number indicated that the up- and downregulated differentially expressed genes may be opposite to the reference gene expression profile; finally, the reference gene expression profile was ranked according to the correlation score [63].

CTRP, which contains sensitivity data for 481 chemicals in 835 CCLs, and the PRISM Repurposing dataset (19Q4, published December 2019), which contains sensitivity data for 1448 chemicals in 482 CCLs, were used to gather drug sensitivity data for CCLs. The AUC value is presented in these two datasets as a measure of drug sensitivity, with lower AUC values suggesting more sensitivity to treatment. Each TCGA sample’s drug sensitivity was assessed using the R package pRRophetic (Version: 4.15-1), which has a built-in ridge regression model that was used to predict the chemotherapy response of clinical samples based on their expression profiles [19, 64]. The AUC values and PRSs were then examined using Spearman correlation to identify compounds with negative correlation coefficients (Spearman r < −0.25 for CTRP and −0.30 for PRISM). Finally, an evaluation of drug response differences between the high-PRS (highest decile) and low-PRS (lowest decile) groups was conducted.

### Statistical analysis

R version 4.0.3 was used for all statistical studies (2020-10-10). The Mann–Whitney U test and the Pearson chi-square test were used to compare continuous and categorical variables between the training cohort and validation cohort, respectively. GSEA was performed to test the TCGA cohort findings with the “c5.go.v7.2.entrez.gmt” gene set using the clusterProfiler R package (Version: 3.18.0; https://bioconductor.org/packages/clusterProfiler/) [65]. Using the meta R package (Version: 4.15- 1; https://cran.r-project.org/web/packages/meta/index.html), a meta-analysis was performed to assess the prognostic significance of the gene signature across all datasets. The primary predictive variables of OS were identified using multivariate and univariate Cox regression models (P < 0.05). The nomogram and calibration curve were plotted using the rms R package. The log-rank test was used to compare Kaplan–Meier survival curves. The volcano plot and heatmap were created using the ggplot2 R package. Pearson’s r correlation and Spearman’s rank-order correlation were used to calculate correlations between two continuous variables. The missing AUC values were imputed using K-nearest neighbor (KNN) imputation. P < 0.05 was regarded statistically significant.

### Data availability statement

The original contributions presented in the study are included in the article/Supplementary Material. Further inquiries can be directed to the corresponding author.

## Supplementary Material

Supplementary Figure 1

Supplementary Tables 1 and 2

Supplementary Table 3
