# Supplementary material for: A novel pyroptosis-related signature predicts prognosis and response to treatment in breast carcinoma
Source: Aging (Albany NY). 2022 Jan 27;14(2):989–1013. doi: 10.18632/aging.203855 (PMC8833126; doi:10.18632/aging.203855)
Supplement: Supplementary Figure 1 [file aging-14-203855-s001.pdf]

SUPPLEMENTARY FIGURE

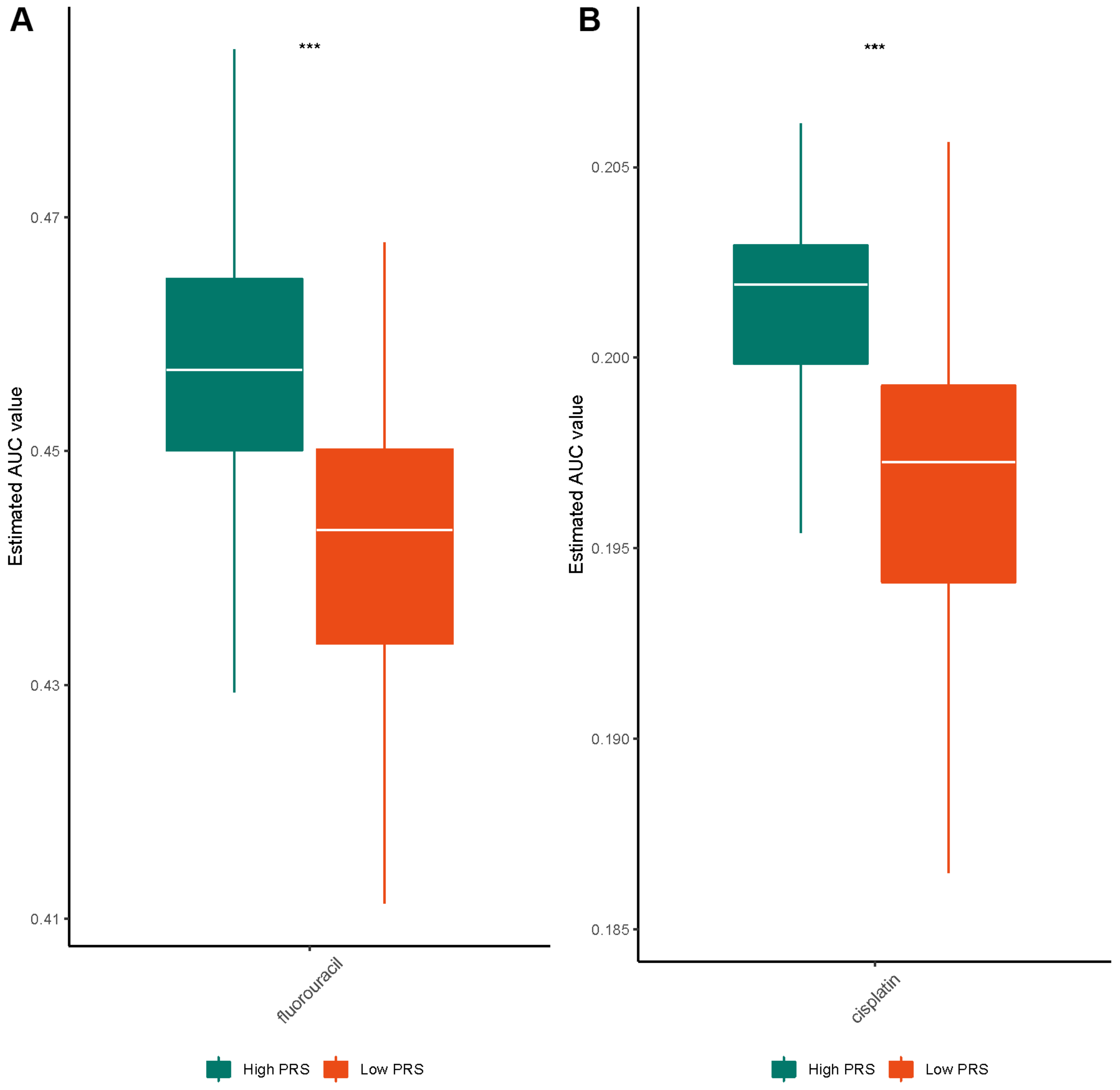

Supplementary Figure 1. Response to fluorouracil and cisplatin of high- and low-IRS patients. The results of widely used chemotherapy drugs, fluorouracil (**A**) and cisplatin (**B**) response analysis between the high PRS and low PRS groups. The low-risk populations had lower AUC values, indicating greater sensitivity to the two common chemotherapeutic agents.
