# Supplementary material for: A novel pyroptosis-related signature predicts prognosis and response to treatment in breast carcinoma
Source: Aging (Albany NY). 2022 Jan 27;14(2):989–1013. doi: 10.18632/aging.203855 (PMC8833126; doi:10.18632/aging.203855)
Supplement: Supplementary Tables 1 and 2 [file aging-14-203855-s002.pdf]

SUPPLEMENTARY TABLES

Supplementary Table 1. 55 pyroptosis-related candidate genes.

Gene symbol	Description	Category
AIM2	Absent In Melanoma 2	Protein Coding
ANO6	Anoctamin 6	Protein Coding
APIP	APAF1 Interacting Protein	Protein Coding
CAMP	Cathelicidin Antimicrobial Peptide	Protein Coding
CASP1	Caspase 1	Protein Coding
CASP3	Caspase 3	Protein Coding
CASP4	Caspase 4	Protein Coding
CASP5	Caspase 5	Protein Coding
CASP8	Caspase 8	Protein Coding
CPTP	Ceramide-1-Phosphate Transfer Protein	Protein Coding
CTSG	Cathepsin G	Protein Coding
DDX3X	DEAD-Box Helicase 3 X-Linked	Protein Coding
DHX9	DExH-Box Helicase 9	Protein Coding
EEF2K	Eukaryotic Elongation Factor 2 Kinase	Protein Coding
ELAVL1	ELAV Like RNA Binding Protein 1	Protein Coding
FADD	Fas Associated Via Death Domain	Protein Coding
FGF21	Fibroblast Growth Factor 21	Protein Coding
FOXO3	Forkhead Box O3	Protein Coding
GBP1	Guanylate Binding Protein 1	Protein Coding
GBP5	Guanylate Binding Protein 5	Protein Coding
GJA1	Gap Junction Protein Alpha 1	Protein Coding
GSDMA	Gasdermin A	Protein Coding
GSDMB	Gasdermin B	Protein Coding
GSDMC	Gasdermin C	Protein Coding
GSDMD	Gasdermin D	Protein Coding
GSDME	Gasdermin E	Protein Coding
GZMA	Granzyme A	Protein Coding
GZMB	Granzyme B	Protein Coding
HDAC6	Histone Deacetylase 6	Protein Coding
HMGB1	High Mobility Group Box 1	Protein Coding
IL18	Interleukin 18	Protein Coding
IL1B	Interleukin 1 Beta	Protein Coding
IL36B	Interleukin 36 Beta	Protein Coding
IL36G	Interleukin 36 Gamma	Protein Coding
IRF3	Interferon Regulatory Factor 3	Protein Coding
MALT1	MALT1 Paracaspase	Protein Coding
MKI67	Marker Of Proliferation Ki-67	Protein Coding
MST1	Macrophage Stimulating 1	Protein Coding
NAIP	NLR Family Apoptosis Inhibitory Protein	Protein Coding
NLRC4	NLR Family CARD Domain Containing 4	Protein Coding
NLRP1	NLR Family Pyrin Domain Containing 1	Protein Coding
NLRP3	NLR Family Pyrin Domain Containing 3	Protein Coding
NLRP7	NLR Family Pyrin Domain Containing 7	Protein Coding
NLRP9	NLR Family Pyrin Domain Containing 9	Protein Coding
NR1H2	Nuclear Receptor Subfamily 1 Group H Member 2	Protein Coding

P2RX7	Purinergic Receptor P2X 7	Protein Coding
PARP1	Poly(ADP-Ribose) Polymerase 1	Protein Coding
PYCARD	PYD And CARD Domain Containing	Protein Coding
SQSTM1	Sequestosome 1	Protein Coding
STING1	Stimulator Of Interferon Response CGAMP Interactor 1	Protein Coding
STK4	Serine/Threonine Kinase 4	Protein Coding
TET2	Tet Methylcytosine Dioxygenase 2	Protein Coding
TP53	Tumor Protein P53	Protein Coding
TREM2	Triggering Receptor Expressed On Myeloid Cells 2	Protein Coding
ZBP1	Z-DNA Binding Protein 1	Protein Coding

Supplementary Table 2. 15 pyroptosis-related gene-based signature.

	exp(coef)	coef
NLRC4	1.62847457	0.48764373
GSDMC	1.298640561	0.261317995
DHX9	1.097058186	0.092632221
FOXO3	1.081542489	0.078388253
IL18	0.827962551	-0.188787354
GJA1	0.8021137	-0.22050491
TP53	1.19988773	0.182227994
FGF21	1.141209354	0.132088537
GBP1	0.656979238	-0.420102863
GZMB	0.708450665	-0.344674856
MST1	0.772059504	-0.258693655
IRF3	1.456089864	0.375754667
IL36B	1.098408554	0.093862364
IL36G	0.852422168	-0.159673372
ANO6	1.324949323	0.281374212
